# Adipose-Derived Cells (Stromal Vascular Fraction) Transplanted for Orthopedical or Neurological Purposes: Are They Safe Enough?

**DOI:** 10.1155/2016/5762916

**Published:** 2016-09-06

**Authors:** Katarzyna Siennicka, Aleksandra Zolocinska, Karolina Stepien, Natalia Lubina-Dabrowska, Marzena Maciagowska, Ewa Zolocinska, Anna Slysz, Renata Piusinska-Macoch, Slawomir Mazur, Urszula Zdanowicz, Robert Smigielski, Adam Stepien, Zygmunt Pojda

**Affiliations:** ^1^Department of Regenerative Medicine, Maria Sklodowska-Curie Memorial Cancer Center and Institute of Oncology, Roentgena 5, 02-781 Warsaw, Poland; ^2^Department of Orthopedics, Carolina Medical Center, Pory 78, 02-001 Warsaw, Poland; ^3^Department of Neurology, Military Institute of Medicine, Szaserow 128, 04-141 Warsaw, Poland

## Abstract

Although mesenchymal stem cells are used in numerous clinical trials, the safety of their application is still a matter of concern. We have analysed the clinical results of the autologous adipose-derived stem cell treatment (stromal vascular fraction (SVF) containing adipose-derived stem cells, endothelial progenitors, and blood mononuclear cells) for orthopedic (cartilage, bone, tendon, or combined joint injuries) and neurologic (multiple sclerosis) diseases. Methods of adipose tissue collection, cell isolation and purification, and resulting cell numbers, viability, and morphology were considered, and patient's age, sex, disease type, and method of cell administration (cell numbers per single application, treatment numbers and frequency, and methods of cell implantation) were analysed and searched for the unwanted clinical effects. Results of cellular therapy were compared retrospectively to those obtained with conventional medication without SVF application. SVF transplantation was always the accessory treatment of patients receiving “standard routine” therapies of their diseases. Clinical experiments were approved by the Bioethical Medical Committees supervising the centers where patients were hospitalised. The conclusion of the study is that none of the treated patients developed any serious adverse event, and autologous mesenchymal stem (stromal) cell clinical application is a safe procedure resulting in some beneficial clinical effects (not analysed in this study).

## 1. Introduction

Mesenchymal stem (stromal) cells (MSCs) are widely used for both allogeneic and autologous transplantations; recently over 500 clinical trials have been registered in https://clinicaltrials.gov. The main sources of MSCs are bone marrow [1] or adipose tissue [2]. The latter source makes possible collection of higher cell numbers [3], which allows avoiding the cell expansion in vitro prior to the transplantation. Autologous MSC transplantation is used for the wide spectrum of indications, mostly in orthopedics [4], neurology [5], and esthetic medicine [6]. Our experience combines the adipose-derived mesenchymal stem cell (ASC) applications in orthopedic or neurologic patients. In both categories, the immunomodulating effect and/or local modulation of in situ factors responsible for wound healing are exploited, rather than the capacity of ASCs to produce by themselves the progeny differentiating into bone [7], cartilage [8], tendon [9], nerve [10], or muscle cells [11]. The importance of problem of autologous ASC treatment safety is minimized by the authors, if the treatment does not include oncologic patients. In our opinion, however, even numerous clinical trials producing data collected from nonhomogenous experiments performed according to highly variable protocols and on not so numerous groups of patients should be treated with high degree of caution. We have analysed our data collected from 145 patients treated with autologous ASC looking for any sign of unwanted side effects.

## 2. Materials and Methods

### 2.1. Patients

**Figure 1:**
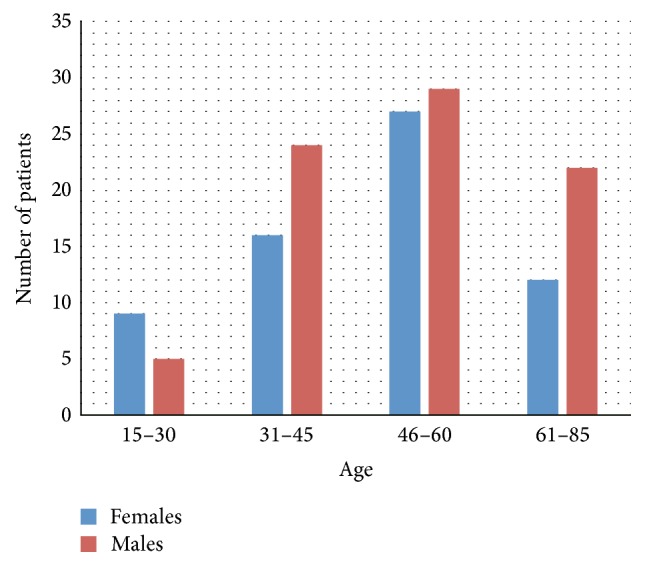
Age and sex distribution of total numbers of patients treated with ASC.

**Figure 2:**
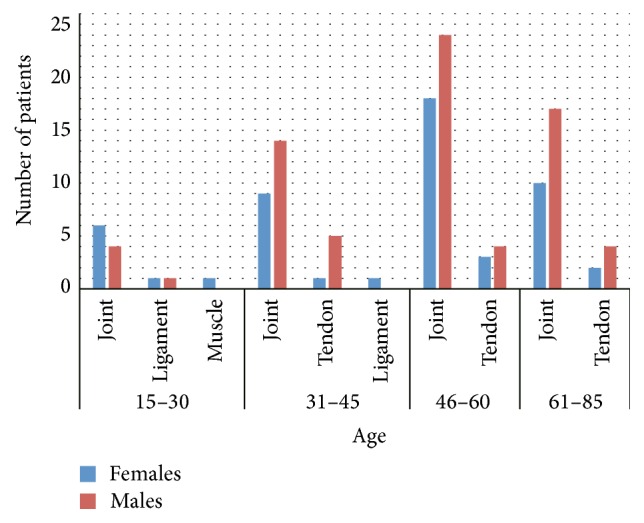
Age and sex distribution of patients injected with ASC for joint, ligament, tendon, or muscle injury.

**Figure 3:**
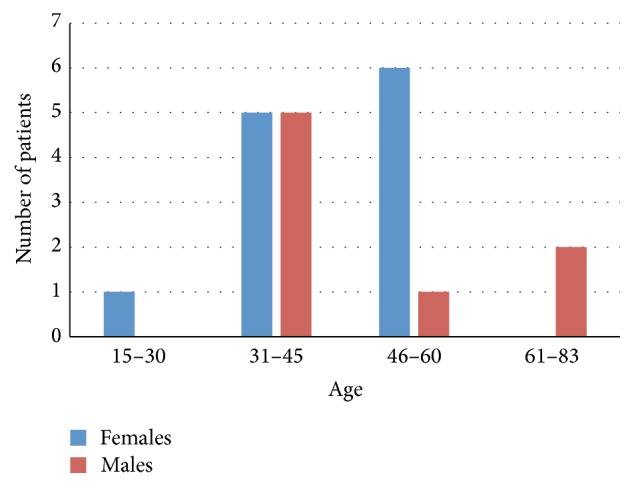
Age and sex distribution of patients treated intrathecally with ASC for multiple sclerosis (MS) disease.

Total number of 145 patients (80 males and 65 females) of age between 15 and 85 years (Figure 1) were treated with stromal vascular fraction (SVF) cells isolated from their adipose tissue collected by Coleman technique or body jet liposuction. Sex and age distribution of the group of 125 patients treated for orthopedic injuries including joints (bone or cartilage injuries), muscle, tendons, and ligaments are presented in Figure 2, whereas 20 patients treated by intrathecal cell injections for multiple sclerosis (MS) disease are presented in Figure 3.

### 2.2. Adipose Tissue Collection and SVF Isolation and Purification

Adipose tissue was collected from abdominal and/or femoral regions under local anesthesia according to Coleman technique (neurological patients) or body jet liposuction (orthopedic patients). Briefly, we have the following: (1) Method according to Coleman [12] was based on the application of syringe with the special collection needle. Prior to liposuction, 300 mL of sterile saline solution supplemented with epinephrine and lignocaine was distributed evenly in collection site by needle injection, and 100–200 mL of fat was subsequently aspirated and processed. (2) Body jet liposuction was performed according to the manufacturer's setting following the sterilisation of machine's collection chamber. Resulting adipose tissue (200–300 mL) was subsequently processed similarly to previously described. Time between adipose tissue collection and processing did not exceed 2 h.

SVF cells were isolated from adipose tissue by collagenase from* Clostridium histolyticum* (Sigma-Aldrich) digestion and gravity separation. PBS- (Life Technologies) diluted fat was treated with 0.025% collagenase solution in 37°C for 1.5 h, and resulting mixture was diluted with PBS + 2% human albumin (KEDRION) and centrifuged (400 g, 10 min) until phase separation. Cell pellet was resuspended in 5% human albumin solution in physiological salt solution for patient injections or control-rate frozen in cryoprotectant containing 5% human albumin and 10% DMSO in hydroxyethyl starch (TETRASPAN, 6% HES 130/0.42, B/Braun) and stored in liquid nitrogen temperature (−196°C).

Routine tests were performed before freezing and after thawing and included sterility control (BACTEC, Bact/Alert, Becton Dickinson, blood agar, Columbia agar, and BHI agar), cell number count (Burker chamber and Sysmex analysis), and viability testing (fluorescence microscopy, acridine orange plus ethidium bromide staining). Infected samples were immediately discarded. Selected samples of SVF cells and adherent fraction cells (ASCs) were FACS analysed for surface markers presence (CD29, CD31, CD34, CD45, CD73, CD90, CD105, Lin1, CXCR4, and HLA-DR), in vitro proliferation potential, and differentiation capacity into adipo-, osteo-, and chondrogenic lineages. Clonality of SVF cells was analysed by CFU-F testing.

### 2.3. Clinical Application of SVF: Orthopedics

125 patients were enrolled in the study. The criteria were recent injury of knee or elbow joint, full or partial mechanical break of tendon or ligament and subsequent surgical repair, or osteoarthritis and chondromalacia. In one case, the surgery of the quadriceps muscle having distal attachments disconnected was performed, assisted with ASC intramuscular treatment. The main exclusion criteria were coexisting cancer disease or pregnancy. Cell suspension was injected directly into muscle, tendon, or ligament or into joint cavity. Cells were introduced into knee joint cavity in 5 doses, average number of 6.5 × 10^6^ cells/5 mL per dose in monthly intervals, and for tendon or ligament repair 3 injections of 7 × 10^6^ cells/1.5 mL per dose in monthly intervals were made. In case of muscle reconstruction, 2.5 × 10^7^ cells suspended in 50 mL of saline were given immediately during reconstruction, followed by 6 injections of 6.0 × 10^6^ cells in monthly intervals.

### 2.4. Clinical Application of SVF: Neurology (Multiple Sclerosis)

15 patients with relapsing-remitting MS form and 5 patients with secondary progressive MS form were enrolled in the study. The main exclusion criteria were coexisting cancer disease and pregnancy. Autologous SVF cells were injected in order to exploit the ASC capability to attenuate the activity of patients immune system against their brain neural cells [13]. Average numbers of 14.2 × 10^6^ cells (females) and 12.8 × 10^6^ cells (males) suspended in 3 mL of saline were injected intrathecally upon enrollment; injections were repeated after 3 and 6 months.

Clinical experiments were approved by the Bioethical Medical Committees supervising the centers where patients were hospitalised.

## 3. Results and Discussion

### 3.1. Results

**Figure 4:**
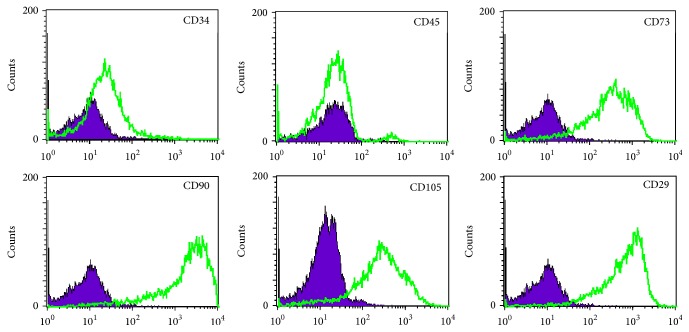
FACS analysis results of cells enzymatically isolated from adipose tissue.

Average cell content per 1 mL of adipose tissue was 8.0 × 10^6^, total cell number per collection was 6.63 × 10^7^, and cell viability measured after 2 h storage (23°C) following isolation was >81%. Analysis of cell surface markers confirmed that SVF cell suspension consists of the mixture of peripheral blood cells, mesenchymal stem cells, and endothelial progenitor cells. The percentages of cell fractions, measured by morphology analysis, hemocytometer (Sysmex) measurement, and flow cytometry, were highly variable and not related to donor sex or age. The percentage of adherent cells was 8–22% and that of mature blood cells (lymphocytes, macrophages, and granulocytes) was 22–40%, and cells of endothelial lineage (CD31+ or CD34+/CD45−) did not exceed 17%. Among the cell surface markers detected in adherent cell population were those specific for MSC: CD73, 91.9%, CD105, 76.2%, and CD90, 93.3% (Figure 4). Presence of CD34 marker with concomitant absence of hematopoietic marker CD45 suggests the limited percentage of EPC cells in SVF; the maximum percentage of EPC in sample was 12.4%, whereas average frequency per sample was 3.35%. Adherent cell population in contrast to SVF was CD31-negative.

**Figure 5:**
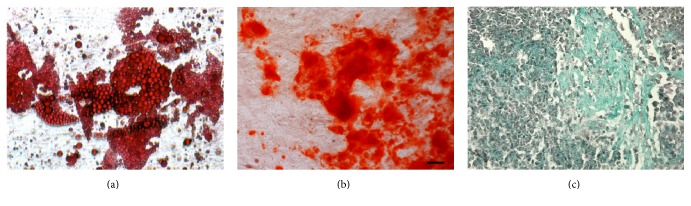
In vitro adipogenic (a), osteogenic (b), and chondrogenic differentiation of adipose-derived adherent cells.

Adherent cells were able to differentiate in vitro into adipo-, osteo-, and chondrogenic lineages (Figure 5), the capacity which, along with their surface markers, confirms their mesenchymal stem cell origin [14, 15]. CFU-F frequency was 6.8%.

The therapeutic effect of ASC treatment has yet to be evaluated by us following longer observation time, and it is out of the scope of the present article. In general, all patients treated with ASC as with the additional treatment supplementing the routine therapy either show some improvement in their clinical status or do not differ from the control group receiving conventional therapy only.

### 3.2. Adverse Events

Following 48 hr monitoring of patient's status after cell transplantation (or longer, depending on the need for the hospitalisation), the observation time was 2.5 years (6 months–3.3 years), and 145 patients were treated with ASCs receiving total number of 480 autologous ASC injections. We did not observe any serious adverse effects in whole patients group. The only such effect which could or could not be related to ASCs themselves was observed in 44-year-old female patient receiving ASCs into both knee joint cavities for the treatment of chondromalacia. The patient reported after second ASC injection transient pain and swelling in the region of right knee joint only (she received cell injection into both knees simultaneously and there was no similar reaction in the left knee region). Next two injections given into both knee joints in monthly intervals were uneventful. This patient has shown the final improvement after completion of therapy.

### 3.3. Discussion

The only stem cell treatment which fulfills the criteria of clinical routine is hematopoietic stem cell transplantation. All the “therapies” using nonhematopoietic stem cells (induced pluripotent stem cells [16], embryo cells [17], fetal stem cells [18], or adult stem cells [19]) are still at the experimental stage. The beginnings of stem cell applications for regenerative medicine purpose, dated at the beginning of 21st century, were based on the expectation of tissue reconstruction by transplanted cells themselves. Stem cells were believed, according to in vitro data, to differentiate into tissues needing repair. Several concepts of cell transdifferentiation or plasticity were supporting the ideas of in vivo role of stem cells (including adult cells) which even after intravenous infusion will be able to localize the site of tissue injury, to home there, and to reconstruct injured organ by producing new tissue [20]. Nowadays, the experiments with tissue formation in vivo are at the rather early stages of development. The most popular cell type for current clinical experiments is now mesenchymal stem (stromal) cell (MSC) [21]. Although in vitro these cells may produce progeny belonging to several differentiation lineages (adipo-, osteo-, or chondropoietic [14, 15], but also nerve cells [10], hepatocytes [22], pancreatic Langerhans islets [23], etc.), their therapeutic potential is mostly based on their immunomodulatory functions and paracrine regulatory activity in situ in close vicinity to the injured tissue. We are using MSC isolated from adipose tissue, which, due to high numbers of ASCs available, is the optimum source for MSC for their immediate transplantation [3]. The other source, bone marrow, allows for the collection of lower MSC numbers than adipose tissue [3] and produces the mixed population of MSC and hematopoietic stem cells (HSCs). The use of fresh, noncultured cell population reduces any (minimal) risk of producing in vitro cells with chromosomal abnormalities [24] and allows (in some countries) avoiding expensive procedure of drug registration.

High expectations combined with stem cells used for therapeutic purposes result in their numerous clinical applications, sometimes lacking the necessary scientific background and the knowledge of possible risks and negative consequences. For that reason, any documented information on possible adverse effects of stem cell therapy (or their absence) is still of great importance, adding to the database summarizing pros and contras of experimental therapies.

## 4. Conclusions

This paper summarizes our experience collected from clinical experiments performed in collaborating clinics which test autologous MSCs for their capabilities of paracrine stimulation of repair of musculoskeletal injures and for immunomodulatory function in multiplex sclerosis disease of central nervous system. We report no serious adverse effects in the group of 145 patients receiving in total 480 cell injections into tendons, ligaments, or muscle or intrathecally. The results allow us to conclude that autologous ASC treatment is (at least in clinical situations described above) a safe procedure and does not cause any substantial risk for the patients.

The study was approved by the Local Bioethical Committees of the institutions involved. The Laboratory of Cellular Engineering and Stem Cell Bank are accredited by Ministry of Health for “processing, isolation, and storage of adipose-derived cells”; the hospital is certified by the Ministry of Health for “collection and transplantation of adipose-derived cells.”
